# Energy Loss Index and Dimensionless Index Outperform Direct Valve Planimetry in Low-Gradient Aortic Stenosis

**DOI:** 10.3390/jcm13113220

**Published:** 2024-05-30

**Authors:** Sarah Hugelshofer, Diana de Brito, Panagiotis Antiochos, Georgios Tzimas, David C. Rotzinger, Denise Auberson, Agnese Vella, Stephane Fournier, Matthias Kirsch, Olivier Muller, Pierre Monney

**Affiliations:** 1 Service de Cardiologie, University Hospital of Lausanne (CHUV), 1011 Lausanne, Switzerlandagnese.vella@hcuge.ch (A.V.); stephane.fournier@chuv.ch (S.F.); olivier.muller@chuv.ch (O.M.); 2 Faculty of Biology and Medicine, University of Lausanne (Unil), 1011 Lausanne, Switzerland; david.rotzinger@chuv.ch (D.C.R.); matthias.kirsch@chuv.ch (M.K.); 3 Service de Radiodiagnostic et Radiologie Interventionnelle, University Hospital of Lausanne (CHUV), 1011 Lausanne, Switzerland; 4 Service de Chirurgie Cardiaque, University Hospital of Lausanne (CHUV), 1011 Lausanne, Switzerland

**Keywords:** low-gradient aortic stenosis, echocardiography, multiparametric assessment, dimensionless index, energy loss index

## Abstract

**Background/Objectives:** Among patients with suspected severe aortic stenosis (AS), discordance between effective orifice area (EOA) and transvalvular gradients is frequent and requires a multiparametric workup including flow assessment and calcium-scoring to confirm true severe AS. The aim of this study was to assess direct planimetry, energy loss index (Eli) and dimensionless index (DI) as stand-alone parameters to identify non-severe AS in discordant cases. **Methods:** In this prospective cohort study, we included consecutive AS patients > 70 years with EOA < 1.0 cm^2^ referred for valve replacement between 2014 and 2017. AS severity was retrospectively reassessed using the multiparametric work-up recommended in the 2021 ESC/EACTS guidelines. DI and ELi were calculated, and valve area was measured by direct planimetry on transesophageal echocardiography. **Results:** A total of 101 patients (mean age 82 y; 57% male) were included. Discordance between EOA and gradients was observed in 46% and non-severe AS found in 24% despite an EOA < 1 cm^2^. Valve planimetry performed poorly, with an area under the ROC curve (AUC) of 0.64. At a cut-off value of >0.82 cm^2^, sensitivity and specificity to identify non-severe AS were 67 and 66%, respectively. DI and ELi showed a higher diagnostic accuracy, with an AUC of 0.77 and 0.76, respectively. Cut-off values of >0.24 and >0.6 cm^2^/m^2^ identified non-severe AS, with a high specificity of 79% and 91%, respectively. **Conclusions:** Almost one in four patients with EOA < 1 cm^2^ had non-severe AS according to guideline-recommended multiparametric assessment. Direct valve planimetry revealed poor diagnostic accuracy and should be interpreted with caution. Usual prognostic cut-off values for DI > 0.24 and ELI > 0.6 cm^2^/m^2^ identified non-severe AS with high specificity and should therefore be included in the assessment of low-gradient AS.

## 1. Introduction

Aortic stenosis (AS) is the predominant valvular disease in developed countries and its prevalence increases exponentially with age [1,2]. Moderate-to-severe AS affects 4% of people above the age of 70 [3]. Untreated severe symptomatic AS is associated with a 1-year mortality rate as high as 50% [4]. Since the disease progresses slowly, elderly patients tend to attribute symptoms of fatigue and dyspnea to normal aging. Therefore, regular clinical screening and timely echocardiographic diagnosis are of utmost importance. 

With a better understanding of the natural history of AS, echocardiographic diagnostic criteria significantly evolved over the last 25 years. Maximal transaortic velocity was the first prognostic marker reported [5] but the diagnostic criteria for severe AS currently include three main echocardiographic parameters, namely maximal transaortic velocity > 4 m/s, transaortic mean pressure gradient > 40 mmHg and effective orifice area (EOA) < 1 cm^2^ as assessed by the continuity equation [6,7]. This simple approach has shown limitations, especially in case of low cardiac output states, where transaortic gradients remain low despite severe stenosis. Such discordance may be associated with reduced [8] as well as conserved left ventricular ejection fraction (LVEF)—the latter typically in small ventricles with diastolic dysfunction [9]—and includes 30% to 50% of cases of significant AS [10,11]. 

Current guidelines recommend considering flow status in case of discordant AS and to determine AS as probably non-severe if transaortic flow is normal [12]. In case of low-flow low-gradient AS, a multiparametric approach is recommended to distinguish severe and pseudo-severe AS, with a specific assessment of symptoms, severity of LV hypertrophy, impaired longitudinal LV function, dobutamine stress echo (DSE) and valvular calcium score [12,13,14,15,16]. The diagnostic process of confirming severe AS has therefore become more complex and requires expertise in the interpretation of the many parameters involved. Moreover, the influence of image quality, blood pressure, alignment of Doppler recording, and accuracy in measurement of the left ventricular outflow tract (LVOT) diameter may add further variability and make an accurate AS severity assessment challenging.

On the other hand, clinicians tend to rely on visual impression and personal experience, particularly in cases of discordant parameters. The temptation to consider direct planimetry of the aortic valve opening area, especially when transesophageal echocardiography (TOE) is available, is therefore high. Planimetry from a short-axis aortic valve view integrates measurement of the anatomic valve area and semi-quantitative assessment of valve sclerosis/calcification to provide a direct impression of severity. However, it is not considered appropriate for clinical application in all patients according to the most recent recommendations [7]. Likewise, several other proposed parameters are currently not recommended for clinical use, or only in selected patients. Such secondary parameters include the ratio of the LVOT to aortic time velocity integral, dimensionless index (DI), and energy loss index (ELi). DI prevents misclassification related to inaccurate LVOT diameter measurement and has shown prognostic value in low-gradient AS [17,18]. ELi corrects the calculation of EOA for the size of the ascending aorta, allowing for the phenomenon of pressure recovery, which may be significant in smaller-size patients [19]. This index has shown independent prognostic value in high- and low-gradient AS [2,20]. 

Our hypothesis was that direct planimetry—even assessed using TOE—would have limited performance and that ELi and DI would demonstrate higher discriminative power in the severity assessment of AS. Thus, the primary objective of this study was to assess the accuracy of direct valve planimetry in discriminating between severe and non-severe AS according to the 2021 ESC/EACTS guidelines criteria in a population of patients with an estimated aortic valve area < 1 cm^2^ referred for aortic valve replacement (AVR). The secondary objective was to assess the diagnostic value of DI and ELi as stand-alone parameters of AS severity. 

## 2. Materials and Methods

### 2.1. Study Population

Between March 2014 and December 2017, consecutive patients with possibly severe AS, referred to our tertiary hospital for AVR, were invited to participate in this prospective single-center study. This study was conducted according to the guidelines of the Declaration of Helsinki and approved by the local Ethics Committee (Commission cantonale d’Ethique de la Recherche sur l’Etre humain du Canton de Vaud, Protocole 319/12, accepted on 17 June 2013) and all patients provided written informed consent. 

Inclusion criteria were age > 70 years, EOA < 1.0 cm^2^, and referral for percutaneous or surgical AVR. Exclusion criteria were concomitant severe aortic regurgitation or severe mitral valve disease, history of previous cardiac surgery, hemodynamic instability requiring emergent surgery, active endocarditis, and constrictive pericarditis. As this was an observational study, we decided to include a prospective cohort of at least 100 patients.

### 2.2. Diagnostic Assessment

For each patient, a complete history was obtained including current symptoms, previous medical history, and cardiovascular risk factors. A complete cardiovascular physical examination was performed including the 6-min walk test. Systematic transthoracic and transesophageal echocardiography was performed according to the 2005 guidelines for chamber quantification [21] and the 2009 recommendations for assessment of valve stenosis [7], with arterial blood pressure measured at the time of echocardiographic examination. Standard parameters of AS severity as well as DI and ELi were evaluated in all patients. DI was calculated as the ratio of the LVOT and the transaortic time velocity integral of the systolic Doppler envelope. ELi was calculated as [EOA × A_A_)/(A_A_ − EOA)], where EOA is the aortic valve area calculated by the continuity equation and A_A_ the cross-sectional area of the aorta at the level of the sino-tubular junction. Direct aortic valve planimetry was obtained from either transesophageal (*n* = 98) or high-quality transthoracic (*n* = 3) short axis view of the aortic valve and independently assessed by two cardiologists (S.H., P.M.) with over 10 years of experience in echocardiography. Coronary angiography was performed in 96 patients (95%) as part of the pre-operative assessment. For every patient, alongside echocardiographic and coronary angiography data, the Society of Thoracic Surgery (STS) risk score [22] was systematically calculated. Cardiac computed tomography (CT) with valvular calcium score calculation was available in 70 patients (69%). A high calcium score was defined as >2000 Agatston units (AU) in men and >1200 AU in women [13]. Either DSE or calcium score was performed in all patients with LVEF < 40% and in 17/22 patients (87%) with a LVEF < 50%. 

All patients were treated by surgical or percutaneous AVR and underwent further systematic echocardiographic assessment after 3 months.

### 2.3. Definition of True Severe Aortic Stenosis

In this initially prospective cohort, we applied a posteriori the criteria of the 2021 ESC/EACTS guidelines for the management of valvular heart disease [11] to discriminate between severe and non-severe AS. In low-gradient AS, flow status was measured, and normal-flow low-gradient AS were reclassified as non-severe AS. Among low-flow low-gradient AS, the true severity of AS was ascertained using the proposed multiparametric approach including valvular calcium score where available. Neither direct valve planimetry, DI nor ELi were part of this multiparametric assessment. 

### 2.4. Statistical Analysis 

Continuous variables were described as median values and interquartile ranges, and categorical variables were described as counts and percentages. All baseline continuous and categorical variables were compared (1) between 3 groups (high-gradient AS, low-flow low-gradient AS and normal-flow low-gradient non-severe AS) using the Kruskal–Wallis test and the Chi2-test, respectively, and (2) between 2 groups (severe and non-severe AS) using the Wilcoxon rank-sum test and the Chi2-test, respectively. 

The concordance between the EOA measured by the continuity equation and aortic valve area measured by direct planimetry as well as the inter-observer agreement for direct valve planimetry were assessed by linear regression and Bland–Altman statistics. The coefficient of variation was calculated as the ratio of the standard deviation and the mean value. The diagnostic accuracies of direct planimetry, DI and ELi were analyzed with receiver operator curve (ROC) analysis and the cut-off value associated with the optimal sensitivity and specificity was estimated for each 3 parameters. The ROC analysis was performed on the whole cohort and was repeated on the subgroup of patients with discordant low-gradient AS. A *p* < 0.05 was considered statistically significant. All analyses were performed using STATA 17.0 software (StataCorp, College Station, TX, USA).

## 3. Results 

### 3.1. Study Population

A total of 101 patients were included in this study, all treated with transcatheter (*n* = 69) or surgical (*n* = 32) AVR. Re-analysis of the baseline echocardiographic and cardiac CT characteristics according to 2021 ESC/EACTS guidelines identified 55 patients (54%) with high-gradient severe AS, 22 patients (22%) with low-flow low-gradient severe AS and 24 patients (24%) with normal-flow non-severe AS. Among patients with EOA < 1 cm^2^, discordant low-gradient AS was present in 46% and non-severe AS in 24% of patients according to these new recommendations.

The clinical and imaging characteristics of patients with severe and non-severe AS are compared in Table 1 and Table 2, respectively; a detailed comparison across the three hemodynamic subgroups is presented in Supplementary Tables S1 and S2. The median age was 82 (77–86) years, with a male predominance (57%). Clinical characteristics were comparable between patients with severe and non-severe AS, although patients with severe AS tended to have higher systolic blood pressure and a lower STS risk score. Regarding the imaging data, there was a trend toward smaller LV size in patients with severe AS but the systolic and diastolic function of the LV was similar. The lower degree of AS severity in the non-severe AS group was characterized by significantly higher EOA (0.87 (0.66–0.96) vs. 0.61 (0.52–0.78) cm^2^, *p* = 0.0006), lower transaortic mean gradients (29 (25–32) vs. 46 (36–58) mmHg, *p* < 0.0001) and lower calcium score (1353 (689–2143) vs. 2375 (1910–3710) AU, *p* = 0.0005). Likewise, echocardiographic re-assessment 3 months post AVR identified a lower absolute increase in EOA (+0.62 (0.37–0.82) vs. +0.91 (0.68–1.11) cm^2^, *p* = 0.02) and a lower reduction in transaortic mean gradient (−19 (14–26) vs. −38 (25–50) mmHg, *p* < 0.0001) in patients with non-severe AS.

### 3.2. Agreement between the Continuity Equation and Direct Aortic Valve Planimetry

**Figure 1:**
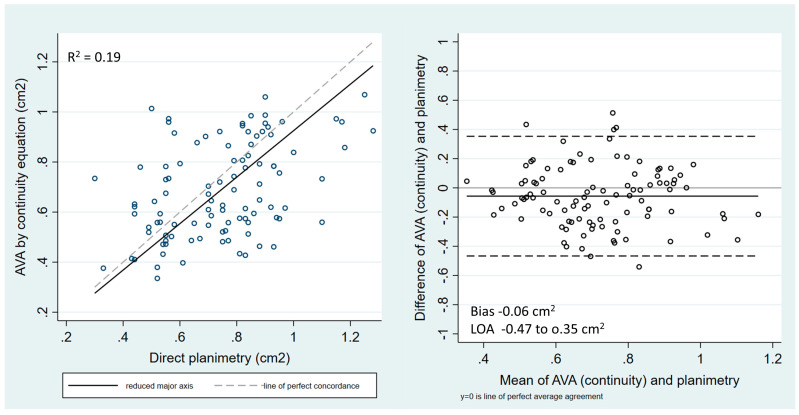
Correlation and concordance between AVA measured by continuity equation and by direct planimetry.

The median EOA for the whole cohort measured by the continuity equation was 0.65 (0.55–0.86) cm^2^ and 0.76 (0.56–0.88) cm^2^ measured by direct planimetry. The correlation between the two parameters was significant but weak, with an r^2^ of 0.19. Bland–Altman statistics identified a bias of 0.06 cm^2^, indicating a systematic overestimation of aortic valve area by planimetry compared to the continuity equation. The standard deviation of the difference was 0.21 cm^2^, corresponding to a coefficient of variation of 29%, and indicated a high variation between the two techniques (Figure 1). Seven patients (7%) had a valve area > 1 cm^2^ as measured by direct planimetry, but two of them (29%) still had severe AS according to the multiparametric assessment. 

In the sub-population of patients with low-gradient AS, the correlation between the two parameters remained weak, with an r^2^ of 0.20. Bland–Altman statistics identified a smaller bias of 0.04 cm^2^ (systematic overestimation of EOA by planimetry) and a similarly high coefficient of variation of 27%.

### 3.3. Direct Valve Planimetry: Inter-Observer Agreement

**Figure 2:**
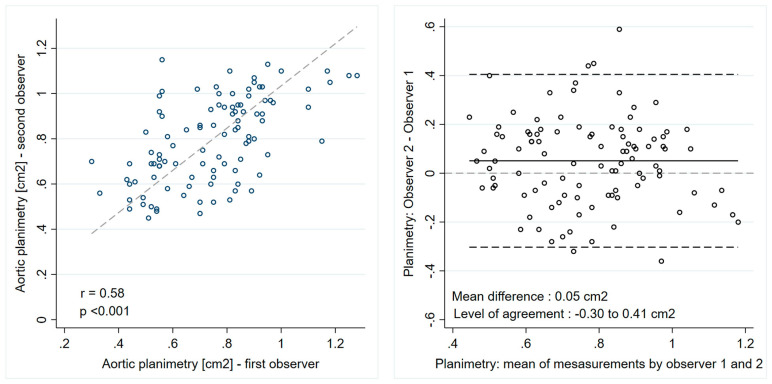
Direct valve planimetry: inter-observer agreement.

The correlation between the measurements performed by two independent observers was low (r^2^ = 0.34, *p* < 0.001). There was a systematic difference of 0.05 ± 0.18 cm^2^ between the two observers and relatively large levels of agreement between −0.30 and 0.41 cm^2^, corresponding to a coefficient of variation of 23% (Figure 2).

### 3.4. Evaluation of Secondary Parameters of AS Severity

The comparison of secondary parameters between non-severe and severe AS are presented in Table 3 (detailed comparison across the three hemodynamic subgroups shown in Supplementary Table S3). Non-severe AS had a slightly but significantly higher EOA as measured by direct planimetry compared to severe AS (0.85 (0.70 −0.90) vs. 0.74 (0.55 −0.86) cm^2^, *p* = 0.04). DI and ELi were markedly and highly significantly larger in non-severe AS compared to severe AS (0.25 (0.21 −0.29) vs. 0.19 (0.16 −0.23), *p* = 0.0001 for DI, and 0.53 (0.41 −0.62) vs. 0.38 (0.33 −0.46) cm^2^/m^2^, *p* = 0.0001 for ELi, respectively). DI showed a significant moderate correlation (r^2^ = 0.38, *p* < 0.001) while ELi showed a significant and high correlation (r^2^ = 0.79, *p* < 0.001) with EOA as assessed by the continuity equation.

**Figure 3:**
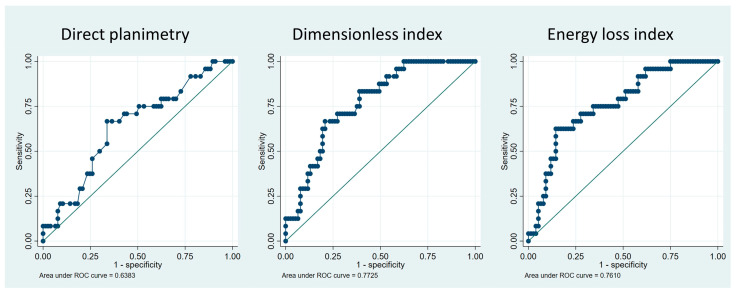
Diagnostic accuracy of secondary parameters for severe aortic stenosis.

The results of the ROC analysis are presented in Figure 3. Direct planimetry provided a modest diagnostic accuracy, with an area under the ROC curve (AUC) of 0.64. With an optimal cut-off value of 0.82 cm^2^, the sensitivity and specificity of direct planimetry in detecting non-severe AS was 67% and 66%, respectively. DI had an AUC of 0.77 (*p* = 0.07 vs. direct planimetry) and, with an optimal cut-off value of 0.24, the sensitivity and specificity for non-severe AS were 67% and 79%, respectively. ELi had an AUC of 0.76 (*p* = 0.04 vs. direct planimetry) and with an optimal cut-off value of 0.52 cm^2^/m^2^, the sensitivity and specificity for non-severe AS were 63% and 86%, respectively. The corresponding sensitivity and specificity at the usual cut-off value of 0.6 cm^2^/m^2^ were 38% and 91%, respectively.

When the same ROC analysis was applied in the subgroup of patients with low-gradient AS, the discrimination performance of planimetry, DI and ELi remained similar, with an AUC of 0.59, 0.71 and 0.74, respectively.

## 4. Discussion

This prospective study including 101 well-characterized patients with possibly severe AS showed a high prevalence of discordant AS, with almost half of the cohort having low-gradient AS. Furthermore, according to the 2021 ESC/EACTS guidelines, one-fourth of these patients were categorized as having non-severe AS after comprehensive assessment. Non-severe AS patients tended to have larger LV volumes, but LVEF and global longitudinal strain were not different in comparison with severe AS. Non-severe AS patients had a higher EOA, lower transaortic gradients and lower calcium score compared with true severe AS patients.

The aortic valve area measured by planimetry was larger than that measured by the continuity equation, with a systematic overestimation of 0.06 ± 0.21 cm^2^. This difference was expected as planimetry measures an anatomic orifice, which is larger than the EOA measured by the continuity equation; indeed, the flow directed to converge toward a narrowed orifice will continue to converge beyond it until surrounding fluid causes divergence [23]. However, the comparison of planimetry and the continuity equation not only revealed a small systematic overestimation, but also a low concordance between the two methods. The low concordance may be related to variability in the EOA as measured by the continuity equation (well-known potential errors in the measurement of the Doppler velocities due to mal-alignment and/or of the LVOT-diameter) but also inaccuracy in direct planimetry of the valve. Even though planimetry was performed on TEE datasets in most patients, measuring errors may occur due to drop-out artefacts in calcified valves, as well as off-axis or below-the-tips valve plane. This is reflected by a relatively high inter-observer variability in valve planimetry results. Consequently, aortic valve area evaluation by planimetry and by the continuity equation may not be used interchangeably for the assessment of AS severity. Indeed, direct valve planimetry as a stand-alone criterion of valve stenosis in our cohort only had a modest diagnostic accuracy, with an AUC of 0.64. With a sensitivity and specificity of only 67% and 66%, respectively, at an optimal cut-off value of 0.82 cm^2^, planimetry appears to be an unreliable tiebreaker in cases of unclear AS severity.

DI is an easily obtained echocardiographic parameter and, compared to the complete continuity equation, its calculation is not affected by errors in the measurement of the LVOT diameter. In a cohort of 488 asymptomatic patients with severe and non-severe AS and normal LVEF, a DI < 0.25 showed a strong prognostic value in terms of mortality or need for AVR over a 5-year follow-up [17]. More recently, in a larger cohort of 755 patients with low-gradient AS and preserved EF, a DI < 0.25 was associated with an increased risk of all-cause mortality over a median follow-up of 61 months [17]. DI appears therefore to be a simple but prognostically attractive maker of AS severity. In our study, DI showed good diagnostic accuracy, with an AUC of 0.77; the optimal discrimination cut-off value was identified at 0.24, a value very close to the prognostic cut-offs of 0.25 defined in previous studies. At this value, DI identified non-severe AS with a specificity of 79%. 

ELi was first described by Garcia et al. [19] as a new index based on the energy loss concept and with the potential of better reflecting AS severity compared to the EOA. Indexing the orifice area to the size of the aorta considers the phenomenon of pressure recovery, which essentially occurs in patients with small diameter aortas. This index is therefore particularly attractive in patients with low-gradient AS and normal EF, as typically seen in elderly women with smaller hearts and diastolic dysfunction. In a post hoc analysis of the Simvastatine and Ezetimibe in Aortic Stenosis study including 498 asymptomatic patients with moderate to severe AS, ELi was an independent predictor of both valvular events and a combined endpoint of mortality and hospitalization over a median follow-up of 4.3 years [20]. Similarly, in a selected population of 379 patients with low-gradient severe AS with preserved EF, 39% of the patients were found to have an ELi > 0.6 cm^2^/m^2^ and were reclassified as moderate AS. Reclassification as moderate AS by ELi was associated with a significantly lower risk of cardiac mortality and need of AVR [2]. In our study, ELi had a significantly higher diagnostic accuracy than valve planimetry in diagnosing severe AS, as an ELi greater than 0.6 cm^2^/m^2^ identified non-severe AS with a high specificity of 91%.

Current guidelines are still mainly based on EOA and transaortic gradients. In cases of discordance between these parameters, flow status needs to be determined, since normal-flow low-gradient AS has a prognosis like that of moderate AS [24,25,26,27] and better than that of low-flow low-gradient or high-gradient AS [24,25,26,28]. However, optimal management remains unclear as other studies reported a benefit of AVR in this population [29]. Although more dedicated studies are needed, the indications for AVR may become broader since normal-flow low-gradient AS seems to rapidly progress towards severe AS [27]. Furthermore, even moderate AS is associated with an adverse prognosis according to large epidemiological data [30]. Several randomized controlled trials are ongoing to study the benefit of transcatheter AVR in patients with moderate AS and LV dysfunction (TAVR UNLOAD; ClinicalTrials.gov: NCT02661451) or cardiac symptoms (PROGRESS; ClinicalTrials.gov: NCT04889872/Evolut EXPAND TAVR II Pivotal Trial; ClinicalTrials.gov: NCT05149755) and will hopefully provide further insight on this conundrum.

Our study has several limitations that deserve comment. Despite a prospective recruitment and thorough characterization of our patients, our cohort remains limited in size and cannot allow a prognostic validation of DI and ELi. Second, valvular calcium score could not be used in all patients for AS severity classification since cardiac CT was only obtained in patients undergoing TAVR. Lastly, our analysis remains largely cross-sectional as all patients underwent AVR and thus the natural history of the disease could not be evaluated. Even though prognostic studies are available for DI and ELi, there are few data on diagnostic and prognostic value of planimetry. Therefore, further studies are needed to better define the value of planimetry.

## 5. Conclusions

In a population of apparently severe AS patients, low-gradient status was observed in almost half of the patients and one in four patients had non-severe AS according to the 2021 ESC/EACTS guidelines. Although frequently considered in clinical practice or during valve board sessions, direct valve planimetry had low reproducibility and was the secondary parameter with the poorest accuracy in discriminating severe from non-severe AS. On the other hand, DI > 0.24 and ELi > 0.52 cm^2^/m^2^ identified non-severe AS with high specificity and had a significantly higher diagnostic accuracy than valve planimetry in diagnosing severe AS. Use of DI and ELi should be considered as part of the multiparametric assessment of low-gradient AS.

## Figures and Tables

**Table 1:** Clinical characteristics according to AS severity in patients with EOA < 1 cm^2^.

		Severe AS (*n* = 77)	Non-Severe AS (*n* = 24)	*p*
Age	y	82 [76–87]	83 [79–86]	0.71
Male sex	*n* (%)	43 (56%)	14 (58%)	0.83
Body mass index	Kg/m^2^	26.4 [23.9–29.4]	26.2 [24.4–28.1]	0.70
Hypertension	*n* (%)	59 (77%)	20 (82%)	0.49
Diabetes	*n* (%)	20 (26%)	6 (25%)	0.92
Hypercholesterolemia	*n* (%)	39 (51%)	14 (58%)	0.51
Smoker	*n* (%)	29 (38%)	11 (46%)	0.48
Systolic blood pressure	mmHg	136 [120–142]	127 [118–139]	0.09
Diast. blood pressure	mmHg	66 [60–75]	70 [61–76]	0.39
Heart rate	bpm	71 [64–85]	72 [64–77]	0.70
Sinus rhythm	*n* (%)	63 (82%)	17 (71%)	0.26
NYHA class III-IV	*n* (%)	47 (61%)	15 (63%)	0.89
6-min walking distance	m	251 [165–373]	243 [160–325]	0.61
STS Score	%	3.3 [2.2–4.7]	4.1 [2.8–8.9]	0.06

**Table 2:** Imaging characteristics according to AS severity in patients with EOA < 1 cm^2^.

		Severe AS (*n* = 77)	Non-Severe AS (*n* = 24)	*p*
LV end-diastolic volume	mL	88 [69–116]	109 [78–132]	0.09
LV ejection fraction	%	59 [48–69]	56 [40–64]	0.14
LV mass index	g/m^2^	110 [92–125]	102 [67–131]	0.29
Mitral E/e’ ratio		13.8 [10.8–18.1]	13.6 [10.5–19.1]	0.84
Global longitudinal strain	%	14.3 [17.3–10.6]	13.4 [16.6–10.0]	0.60
LV outflow tract diameter	mm	20 [19–22]	20 [18–22]	0.47
Stroke volume index	mL/m^2^	33.5 [28.3–43.0]	37.8 [27.2–43.0]	0.93
AVA (continuity equation)	cm^2^	**0.61 [0.52–0.78]**	**0.87 [0.66–0.96]**	**0.0006**
Aortic maximal velocity	cm/s	**430 [362–485]**	**335 [322–367]**	**<0.0001**
Aortic mean gradient	mmHg	**46 [36–58]**	**29 [25–32]**	**<0.0001**
Calcium score (*n* = 70)	AU	**2375 [1910–3710]**	**1353 [689–2143]**	**0.0005**
High calcium score (*n* = 70)	*n*(%)	**45 (92%)**	**6 (29%)**	**<0.001**
Coronary artery disease	*n*(%)	35 (47%)	12 (55%)	0.55

Bold: Significant difference.

**Table 3:** Secondary echocardiographic parameters accordingv to AS severity in patients with EOA < 1 cm^2^.

		Severe AS (*n* = 77)	Non-Severe AS (*n* = 24)	*p*
AVA (continuity equation)	cm²	0.61 [0.52−0.78]	0.87 [0.66−0.96]	0.0006
AVA (planimetry)	cm²	0.74 [0.55−0.86]	0.84 [0.70−0.90]	0.04
Dimensionless index		0.19 [0.16−0.23]	0.25 [0.21−0.29]	0.0001
Energy loss index	cm²/m²	0.38 [0.33−0.46]	0.53 [0.41−0.62]	0.0001

## Data Availability

No external access to data is provided. Patients signed consent only for local utilization of data.

## Supplementary Materials

The following supporting information can be downloaded at: https://www.mdpi.com/article/10.3390/jcm13113220/s1, Supplementary Table S1: Clinical characteristics according to hemodynamic AS group. Supplementary Table S2: Imaging characteristics according to hemodynamic AS group. Supplementary Table S3: Secondary echocardiographic parameters according to hemodynamic AS group.
