# Supplementary material for: Energy Loss Index and Dimensionless Index Outperform Direct Valve Planimetry in Low-Gradient Aortic Stenosis
Source: J Clin Med. 2024 May 30;13(11):3220. doi: 10.3390/jcm13113220 (PMC11173056; doi:10.3390/jcm13113220)
Supplement: Supplementary file 1 [file jcm-13-03220-s001.zip › jcm-3002355-supplementary.pdf]

SUPPLEMENTARY TABLES

Suppl. Table S1: Clinical characteristics according to the hemodynamic AS group.

		All (n=101)	High gradient severe AS (n=55)	Low flow low gradient severe AS (n=22)	Non-severe AS (n=24)	p
Age	y	82 [77-86]	82 [76-87]	82 [76-86]	83 [79-86]	0.86
Male	n (%)	57 (56%)	31 (56%)	12 (55%)	14 (58%)	0.97
Body mass index	Kg/m^2	26.4 [23.9-28.6]	26.6 [24.2-28.4]	26.2 [23.0-30.8]	26.2 [24,4-28.1]	0.92
Hypertension	n (%)	79 (78%)	42 (76%)	17 (77%)	20 (83%)	0.78
Diabetes	n (%)	26 (26%)	13 (24%)	7 (32%)	6 (25%)	0.76
Hypercholesterolemia	n (%)	53 (52%)	29 (53%)	10 (45%)	14 (58%)	0.77
Smoker	n (%)	40 (40%)	20 (36%)	9 (41%)	11 (45%)	0.72
Systolic blood pressure	mmHg	133 [119-142]	138 [126-143]	124 [111-141]	127 [118-139]	0.03
Diast. blood pressure	mmHg	66 [60-75]	64 [59-75]	66 [61-73]	70 [61-76]	0.65
Heart rate	bpm	71 [64-82]	71 [64-85]	71 [64-85]	72 [64-77]	0.90
Sinus rhythm	n (%)	80 (79%)	48 (87%)	15 (68%)	17 (71%)	0.09
NYHA class III-IV	n (%)	62 (61%)	32 (58%)	15 (68%)	15 (63%)	0.71
6-min walking distance	m	248 [160-360]	248 [160-380]	254 [170-365]	243 [160-325]	0.83
STS-Score	%	3.6 [2.3-5.2]	2.9 [2.1-4.6]	3.7 [2.3-7.1]	4.1 [2.8-8.9]	0.06

Suppl. Table S2: Imaging characteristics according to the hemodynamic AS group.

		All (n=101)	High gradient severe AS (n=55)	Low flow low gradient severe AS (n=22)	Non-severe AS (n=24)	p
LV end-diastolic volume	ml	92 [70-117]	86 [69-112]	95 [67-125]	109 [78-132]	0.16
LV ejection fraction	%	58 [47-68]	62 [54-71]	49 [43-68]	56 [40-64]	0.01
LV mass index	g/m^2	108 [89-125]	113 [92-125]	105 [95-126]	103 [67-131]	0.14
Mitral E/e' ratio		13.8 [10.7-18.1]	13.8 [11.1-18.4]	14.0 [9.6-16.1]	13.6 [10.5-19.1]	0.83
Global longitudinal strain	%	14.2 [17.2-10.6]	15.5 [18.4-11.4]	11.2 [14.3-8.3]	13.4 [16.6-10.0]	0.04
LV outflow tract diameter	mm	20 [19-22]	21 [19-22]	20 [19-22]	20 [18-22]	0.36
Stroke volume index	ml/m^2	34.2 [27.8-43.0]	39.9 [29.6-44.8]	28.1 [25.1-29.8]	37.8 [27.2-43.0]	0.0001
AVA (continuity equation)	cm2	0.65 [0.55-0.86]	0.61 [0.50-0.78]	0.64 [0.56-0.78]	0.87 [0.66-0.96]	0.002
Aortic maximal velocity	cm/s	405 [334-469]	454 [428-497]	338 [283-362]	335 [322-367]	0.0001
Aortic mean gradient	mmHg	42 [30-54]	54 [45-63]	30 [18-34]	29 [25-32]	0.0001
Calcium score (n=70)	AU	2117 [1524-3257]	2541 [1768-3939]	2155 [1917-3086]	1353 [689-2143]	0.001
High Calcium score (n=70)	n(%)	51 (72%)	30 (88%)	15 (100%)	6 (29%)	<0.0001
Coronary artery disease	n(%)	47 (49%)	19 (36%)	16 (76%)	12 (55%)	0.006

Suppl. Table S3: Secondary echocardiographic parameters according to the hemodynamic AS group.

		All (n=101)	High gradient severe AS (n=55)	Low flow low gradient severe AS (n=22)	Non-severe AS (n=24)	p
AVA (continuity equation)	cm2	0.65 [0.55-0.86]	0.61 [0.50-0.78]	0.64 [0.56-0.78]	0.87 [0.66-0.95]	0.002
AVA (planimetry)	cm2	0.76 [0.56-0.88]	0.70 [0.54-0.84]	0.76 [0.55-0.91]	0.84 [0.70-0.90]	0.08
Dimensionless index		0.21 [0.17-0.25]	0.18 [0.16-0.22]	0.20 [0.18-0.25]	0.25 [0.21-0.29]	0.0001
Energy loss index	cm2/m2	0.40 [0.33-0.52]	0.37 [0.31-0.44]	0.40 [0.34-0.46]	0.53 [0.41-0.62]	0.0005
